# The physical biogeography of *Fusobacterium nucleatum* in health and disease

**DOI:** 10.1128/mbio.02989-24

**Published:** 2025-03-10

**Authors:** John P. Connolly, Libusha Kelly

**Affiliations:** 1 Department of Systems and Computational Biology, Albert Einstein College of Medicine, Bronx, New York, USA; 2 Department of Microbiology and Immunology, Albert Einstein College of Medicine, Bronx, New York, USA; University of Hawaii at Manoa, Honolulu, Hawaii, USA

**Keywords:** microbial ecology, gut microbiome, oral microbiome, inflammatory bowel disease, metagenomics, genomics

## Abstract

*Fusobacterium nucleatum* (*Fn*) is an oral commensal inhabiting the human gingival plaque that is rarely found in the gut. However, in colorectal cancer (CRC), *Fn* can be isolated from stool samples and detected in metagenomes. We hypothesized that ecological characteristics of the gut are altered by disease, enabling *Fn* to colonize. Multiple genomically distinct populations of *Fn* exist, but their ecological preferences are unstudied. We identified six well-separated populations in 133 *Fn* genomes and used simulated metagenomes to demonstrate sensitive detection of populations in human oral and gut metagenomes. In 9,560 samples from 11 studies, *Fn* population C2 animalis is elevated in gut metagenomes from CRC and Crohn's disease patients and is observed more frequently in CRC stool samples than in the gingiva. Polymorphum, the most prevalent gingival *Fn* population, is significantly increased in Crohn's stool samples; this effect was significantly stronger in male hosts than in female. We find polymorphum genomes are enriched for biosynthetic gene clusters and fluoride exporters, while C2 animalis are high in iron transporters. *Fn* populations thus associate with specific clinical and demographic phenotypes and harbor distinct functional features. Ecological differences in closely related groups of bacteria inform microbiome impacts on human health.

**IMPORTANCE:**

*Fusobacterium nucleatum* is a bacterium normally found in the gingiva. *F. nucleatum* generally does not colonize the healthy gut, but is observed in approximately a third of colorectal cancer (CRC) patient guts. *F. nucleatum*'s presence in the gut during CRC has been linked to worse prognosis and increased tumor proliferation. Here, we describe the population structure of *F. nucleatum* in oral and gut microbiomes. We report substantial diversity in gene carriage among six distinct populations of *F. nucleatum* and identify population disease and body-site preferences. We find the C2 animalis population is more common in the CRC gut than in the gingiva and is enriched for iron transporters, which support gut colonization in known pathogens. We find that C2 animalis is also enriched in Crohn's disease and type 2 diabetes, suggesting ecological commonalities between the three diseases. Our work shows that closely related bacteria can have different associations with human physiology.

## INTRODUCTION

*Fusobacterium nucleatum* (*Fn*) is a gram-negative anaerobe that typically resides in the human oral cavity, primarily in the subgingival plaque (1, 2). *Fn* is thought to live mainly as a commensal, but can act as an opportunistic pathogen, most commonly in periodontal infections (3, 4). *Fn* is of particular interest in colorectal cancer (CRC) due to its identification in metagenomic samples and its subsequent isolation from tumor tissue (5, 6). As *Fn* is not normally found in the gut microbiome, its potential role in causing or aggravating CRC was investigated. Multiple studies have since established that *Fn* occurs in roughly 30% of CRC tumors; when present, it is associated with treatment resistance and poor prognosis (7). Patients' oral microbiota is thought to seed their tumors with *Fn*. There is known genomic diversity among *Fn* in the oral microbiota, with multiple proposed populations: *animalis*, *polymorphum*, *nucleatum*, and *vincentii* (8, 9). A recent study has proposed that *animalis* consists of two clades, C1 and C2, and demonstrated that C2 is enriched in CRC (9). To date, most studies do not consider population-level differences in *Fn* and potential implications for health and disease. Studies in other microbial ecosystems have shown that closely related microbial populations can have phenotypic differences related to key ecosystem pressures (10–12). Population-level differences in a human colonizing bacterial group such as *Fn* could reflect differences in host physiology in health and disease. To our knowledge, no study has compared the prevalence of *Fn* populations in the mouth to their prevalence in CRC or examined *Fn* populations in gut diseases besides CRC.

We propose that analysis of *Fn* populations and their distributions, across body sites and diseases, could reveal differences in ecological parameters that determine how *Fn* interacts with human physiology. Here, we test the hypothesis that diseases with similar microenvironments host similar, ecologically coherent *Fn* populations. We created a horizontal gene transfer (HGT) network of *Fn* and simulated metagenomes of oral and gut environments to empirically determine whether a specific *Fn* population is present in a metagenomic sample. We analyzed approximately 10,000 metagenomic samples from multiple body sites and disease conditions to reveal the clinically relevant, non-uniform distribution of *Fn* populations in body sites and diseases.

## RESULTS

### Available *F. nucleatum* genomes span body sites and geographic locations

We obtained 133 complete *F. nucleatum* genomes from NCBI, excluding anomalous or metagenome-assembled genomes, all at the scaffold level or above. The genomes used were derived from patient samples across different body sites and conditions, including samples from the gut, oral cavity, and urogenital tract (Table 1). Genomes were derived from both healthy and diseased samples, from conditions including CRC, periodontitis, and gingivitis. They included 79 samples from Asia, 54 from the United States, and 3 from Europe (Table 2). The majority of the genomes originated with patients in Korea, China, and the United States.

**TABLE 1:** Site breakdown of genomes included in this analysis^*a*^

Site	Count
Oral	70
Gut	48
Urogenital	3
Sewage	1
N/A	11

^*a*^ N/A indicates that a site was not specified.

**TABLE 2:** Geographic breakdown of genomes included in this analysis^*a*^

Location	Count
Korea	58
USA	54
China	12
UK	1
Netherlands	1
India	1
Denmark	1
N/A	5

^*a*^ N/A indicates that a location was not specified.

### HGT networks of *F. nucleatum* genomes reveal six separate populations

**Fig 1:**
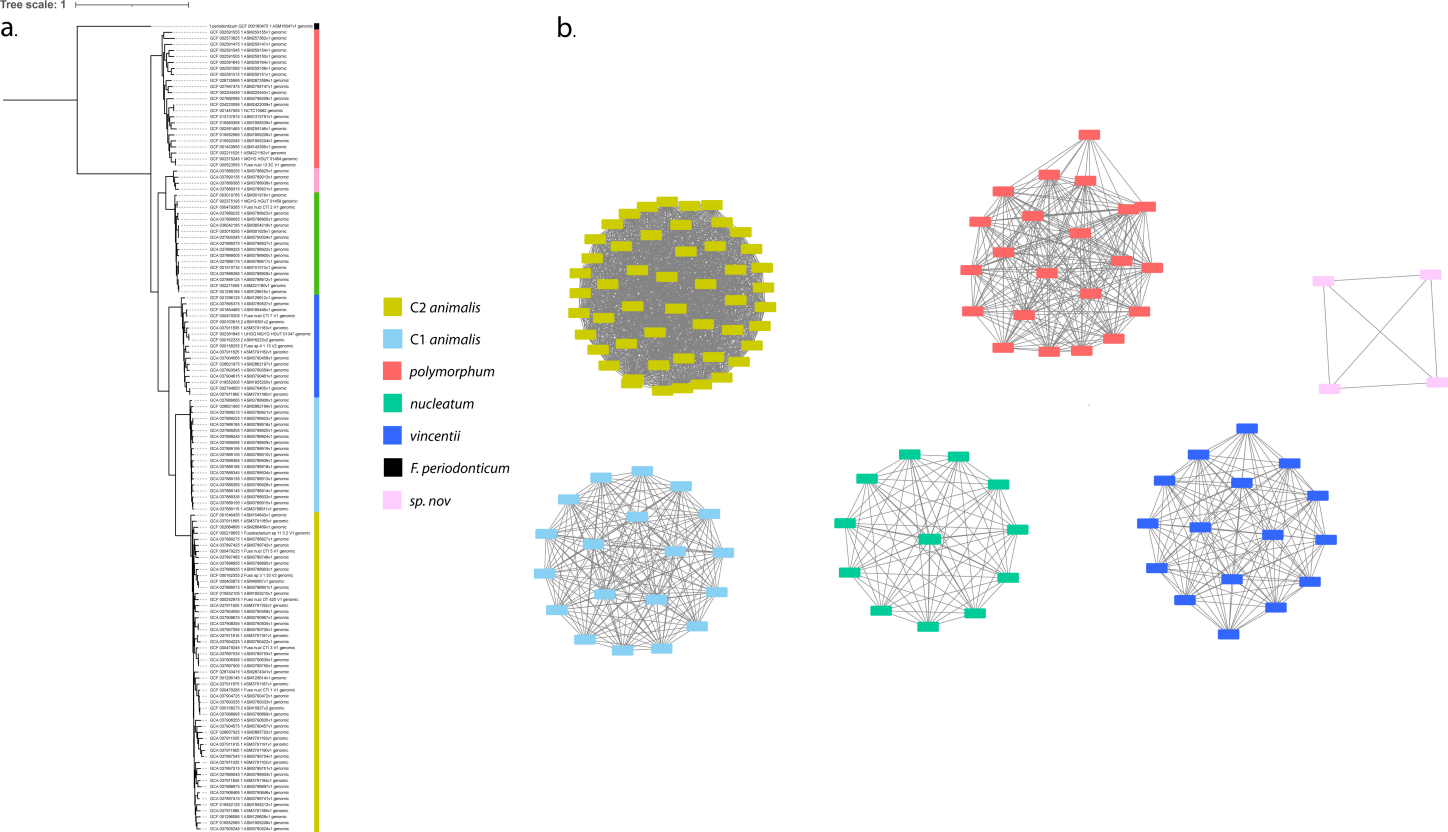
Horizontal gene transfer (HGT) networks of *F. nucleatum* genomes. (a) Core genome phylogeny of 133 *Fn* genomes with one *F. periodonticum* genome as an outgroup. (b) HGT network of *Fn* genomes. Each node reflects a single genome or clonal cluster of genomes, while edges represent the strength of recent HGT between each node pair, shorter edges indicating a stronger relationship. A lack of an edge between two nodes means that the amount of HGT was not significantly different from zero. Colors indicate separate populations. Population labels inferred from previous studies except sp. nov*.* (pink).

We aligned *Fn* genomes pairwise and created a core genome phylogeny using PHAME (13) (Fig. 1a) and a HGT network using the PopCOGenT pipeline (Fig. 1b) (14). We recovered six well-separated populations of bacteria. The populations supported previously proposed subspecies, *nucleatum*, *polymorphum*, and *vincentii*, as well as the recently proposed new *animalis* clades, referred to as C1 and C2 (Fig. 1) (9). Additionally, we found a new population, not previously reported, which appears most closely related to *nucleatum*. We refer to this new population as *Sn* (for *sp. nov*). A plurality of the genomes, 52, clustered with *animalis* C2.

Though each network was highly interconnected, we did not find a single instance of HGT connecting any network to any other, suggesting that the populations inhabit separate ecological niches in the human body. This separation was observed in spite of the fact that all *Fn* populations are commonly found in the human gingiva, supporting the hypothesis that the human body can host closely related, but ecologically distinct populations of bacteria, a phenomenon that has been observed in soil and ocean ecosystems, and is an emerging area of research in human settings (10, 15, 16). To date, most similar investigations of the human microbiota have focused on the strain level rather than the population level (17, 18). Our results suggest that these populations of *F. nucleatum* should more properly be thought of as species than subspecies.

### *F. nucleatum* pangenome functionally differentiates populations

**Fig 2:**
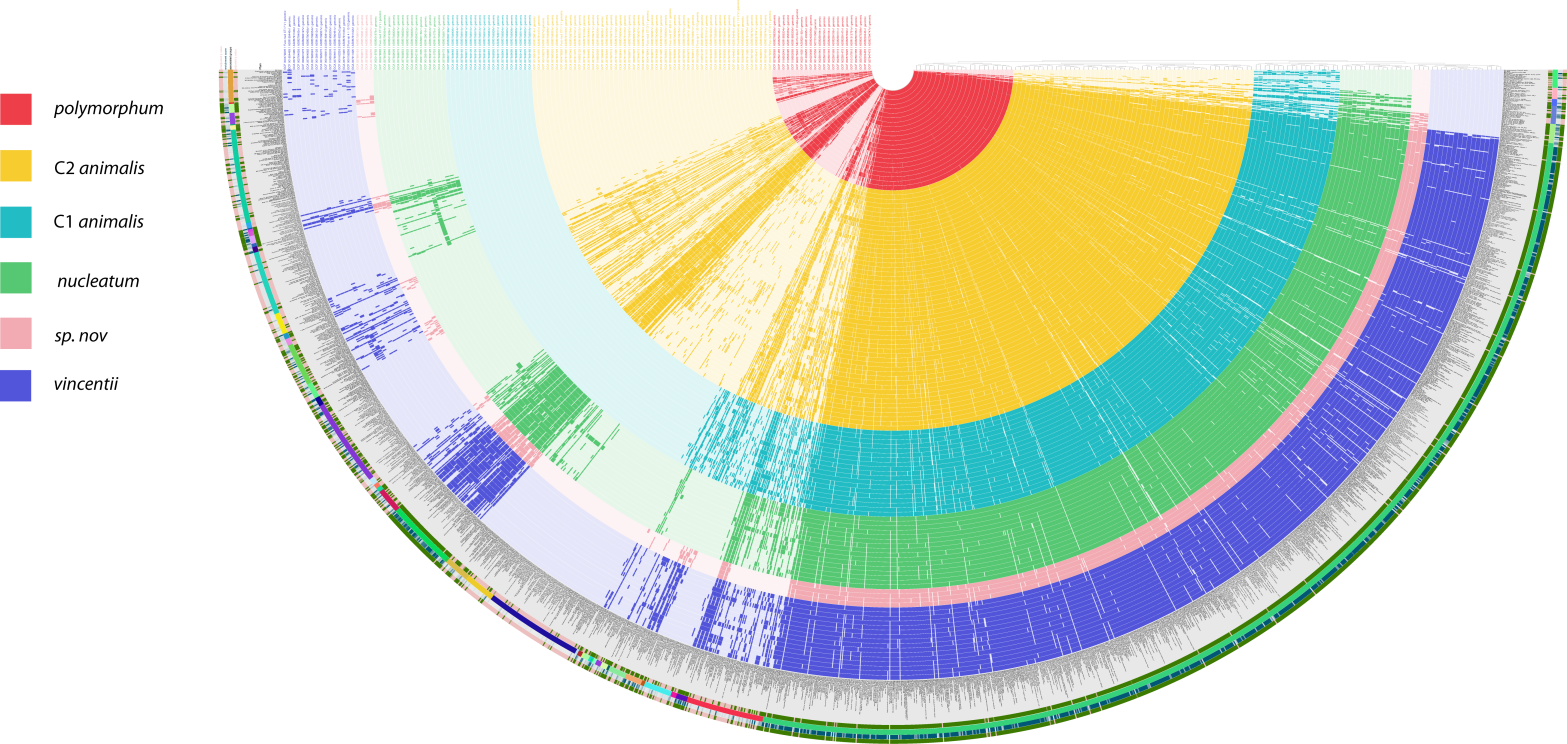
*F. nucleatum* gene clusters in genomes, clustered by presence and absence. 1,425 gene clusters (radial bars) in the genomes were annotated according to protein family, using the Pfam database. The genomes were then clustered according to the presence and absence of protein families, using Ward clustering. To compare these groups to the HGT clusters, we colored each genome with its HGT population assignment.

Having classified the genomes into HGT clusters, we next sought to determine if certain gene functions distinguished each population and if functional analysis supported previous population assignments. We detected 1,425 gene clusters among all genomes using the Anvi'o metagenomics suite and classified the clusters into protein families according to the Pfam database (now InterPro) (19–21). We then sorted the genomes by the presence and absence of each gene cluster. We then colored the genomes by their PopCOGenT assignment and found that the Pfam-defined clusters completely matched the HGT clusters. C1 and C2 *animalis* are adjacent to one another in the pangenome, but C2 possessed a large number of gene clusters that C1 lacked, consistent with the total separation seen in the HGT clusters (Fig. 2).

Previously published phylogenies of *F. nucleatum* have suggested that *vincentii* is as closely related to the *animalis* clades as it is to *nucleatum* (22). However, in this pangenome, *vincentii*, *nucleatum*, and *Sn* (Fig. 2, blue, green, and pink, respectively) all appeared to have similar gene cluster profiles, albeit not identical to one another. Similarly, a relatively small number of gene clusters appeared to distinguish *polymorphum* from the *animalis* clades, despite the fact that they are the most distant groups in published phylogenies. A core genome phylogeny also places *polymorphum* and *animalis* far apart (Fig. 1a) (8, 9).

### Sequence breadth thresholding allows sensitive *F. nucleatum* detection in oral and stool metagenomes

Examining the populations above in metagenomic data sets would support efforts to address the epidemiological relevance of populations. However, no metagenomic tools currently distinguish between the *F. nucleatum* subtypes (23, 24). It was therefore necessary to establish an empirical detection threshold for *F. nucleatum*.

In metagenomics studies, coverage (number of reads assigned to target) is often used to establish bacterial abundance. However, coverage readings can be biased by phages, mobile genetic elements, and resistance cassettes (25). We therefore elected to use sequence breadth (percentage of target genome covered), also known as “detection,” as our metric, because it is less biased by small, high-coverage regions (26, 27). Though sequence breadth is a more reliable means of determining bacterial presence in a metagenome, detection thresholds have not been standardized, and various cutoffs have been used in previous studies, including 50%, 25%, and 10% (19, 26, 28). These thresholds have been determined on more or less arbitrary grounds. An empirically grounded solution was developed in imGLAD, a tool that uses simulated metagenomes to determine the threshold for 95% probability of detection (26).

**Fig 3:**
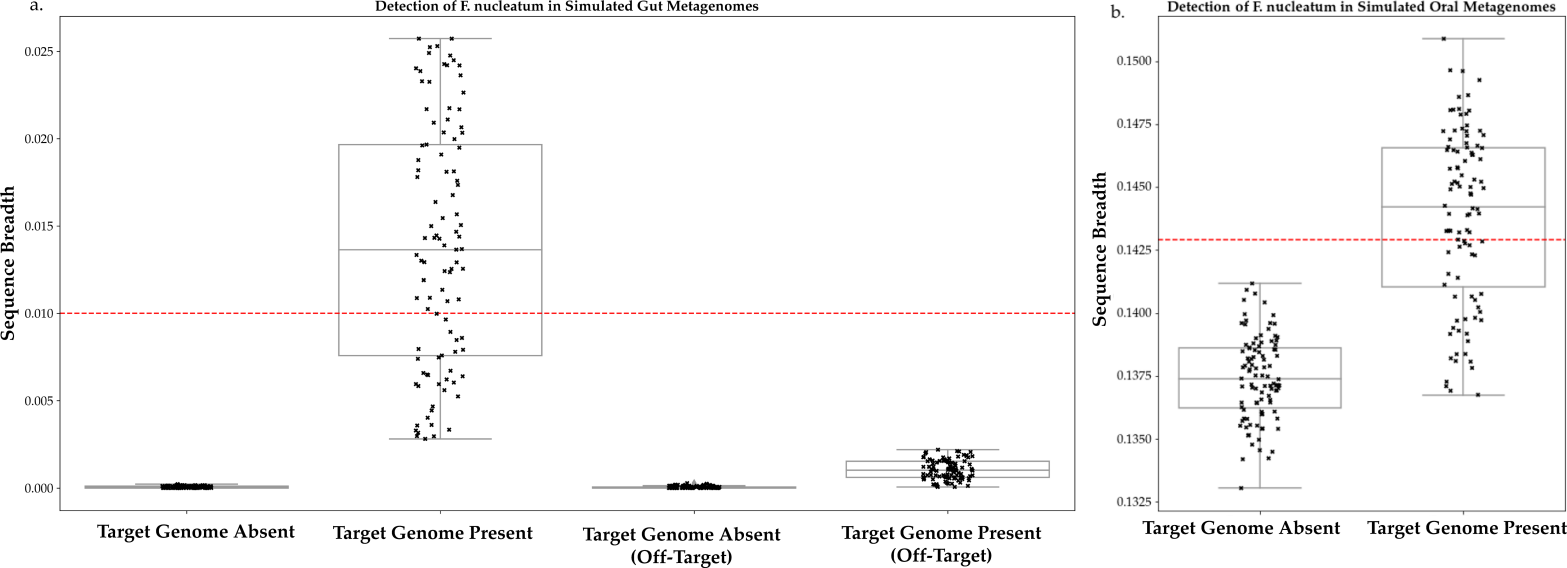
Sequence breadth measurements in simulated metagenomic reads. Simulated gut and oral metagenomes were created by sampling reads from the genomes of common gut and gingival organisms, respectively. *Fn* reads were spiked into 500 of these simulated metagenomes for each environment and compared to 500 without *Fn* reads. The sequence breadth of the target *Fn* genome in each simulation was recorded, and the groups were compared for each environment via logistic regression. The red dashed line indicates sequence breadth for 95% confidence interval in target genome presence for each environment. (a) Sequence breadth graph of simulated gut metagenomes. (b) Sequence breadth graph of simulated oral metagenomes.

Using imGLAD, we generated 1,000 simulated metagenomes, 500 positive controls (containing *F. nucleatum*), and 500 negative controls (no *F. nucleatum*). We separated oral and gut microbiota samples and performed thresholding for each body site using background reads from the appropriate site (Fig. 3). These experiments were performed using each population; the data from the C2 *animalis*-targeted simulations are shown here. Thresholds were similar across populations.

In the gut microbiome, we found that a threshold of 0.01 was sufficient to distinguish positive samples from negative samples, corresponding to ~22 kb (Fig. 3a). We attribute this difference to the absence of *Fusobacteriota* among common resident gut bacteria (29).

By contrast, in the simulated oral metagenomes, we found that a sequence breadth of 0.1429, or ~14.3% of the target genome, was sufficient for 95% confidence interval (CI) in target presence (Fig. 3b). The *F. nucleatum* genome is 2.17–2.4 Mb in length, meaning that we need to recover 310–343 kb of a specific genome from a given oral metagenome for 95% CI in detection.

### *F. nucleatum* population distributions differ by body site

Using the cutoffs generated above, we examined the presence of each *Fn* population in metagenomic data. We sourced 5,840 metagenomes from stool and 3,720 metagenomes from the oral cavity. The data included samples from patients with CRC (*n* = 252), Crohn’s disease (*n* = 596), ulcerative colitis (*n* = 371), type 2 diabetes (T2D) (*n* = 162), periodontitis (*n* = 48), and healthy volunteers (*n* = 7,337). For each metagenomic sample, we determined the sequence breadth for each genome using the Anvi'o metagenomics suite. We compared measurements to the cutoffs calculated above to determine the presence and absence of each population.

**Fig 4:**
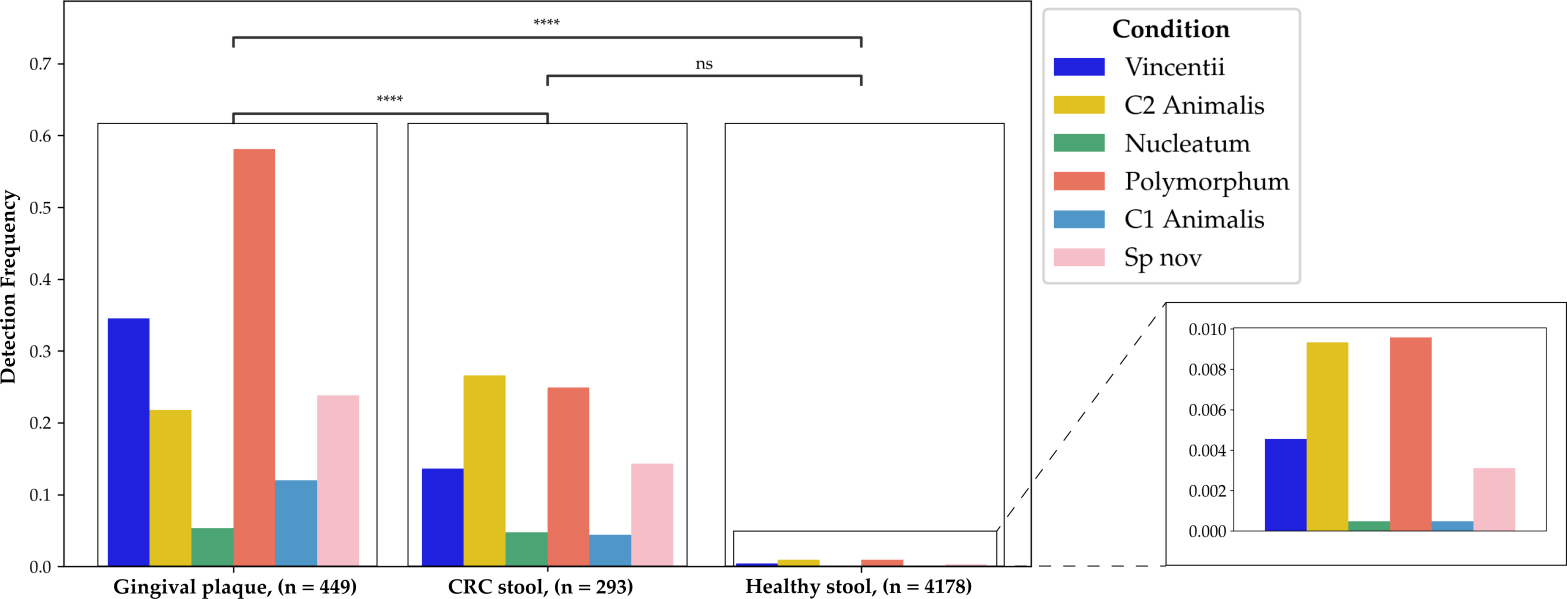
Frequency of *F. nucleatum* population detection by body site and condition using the detection thresholds calculated previously (0.01 sequence breadth for the gut, 0.1429 for the gingiva), *F. nucleatum* detection frequency was analyzed in 6,289 metagenomes from healthy stool, diseased stool, and healthy gingiva. Any sample with sequence breadth above the 95% confidence interval threshold was considered a positive detection. Distributions were compared via chi-squared test of independence. Asterisks indicate *P* < 0.0001, after Bonferroni correction.

In the sub- and supra-gingival plaque, we found that *polymorphum* and *vincentii* were the most common populations in both healthy patients and those with infections, appearing in more than 50% and 30% of gingival plaque samples, respectively (Fig. 4). The previously undescribed *Sn* was roughly as common as C2 *animalis* in the gingiva. C1 *animalis* and *nucleatum* were the least common gingival populations. Our data set included 2,682 saliva samples from multiple studies. However, we did not detect any *Fn* population in saliva, which is consistent with *Fn*’s designation as a gingival plaque resident (30).

To determine if any populations were of special relevance to the gut microbiome, we compared the gingival populations to those found in stool. We compared stool from CRC patients and healthy volunteers (Fig. 4). The distribution of populations in stool did not mirror that of the gingiva (chi-squared test of independence, *P* < 2e−21).

Stool from CRC patients specifically, and unhealthy patients broadly, showed the same population distribution, with C2 *animalis* and *polymorphum* being the first and second most commonly found, respectively. The distributions were not significantly different from healthy to CRC stool, although the frequency was higher in disease samples (Fig. 4).

The absolute frequency of all populations was lower in stool than in the gingiva, with the notable exception of C2 *animalis*, which was found in CRC stool at a higher frequency than in the gingiva (Fig. 4).

### C2 *animalis* is significantly elevated in CRC and T2D

Metagenomic coverage is commonly used as a proxy for how abundant a specific species is in a sample (31). As discussed above, we focused on sequence breadth, rather than coverage, in order to limit false positive detection. To assess how abundant populations are during different disease states, we compared sequence breadth measurements across populations and conditions.

**Fig 5:**
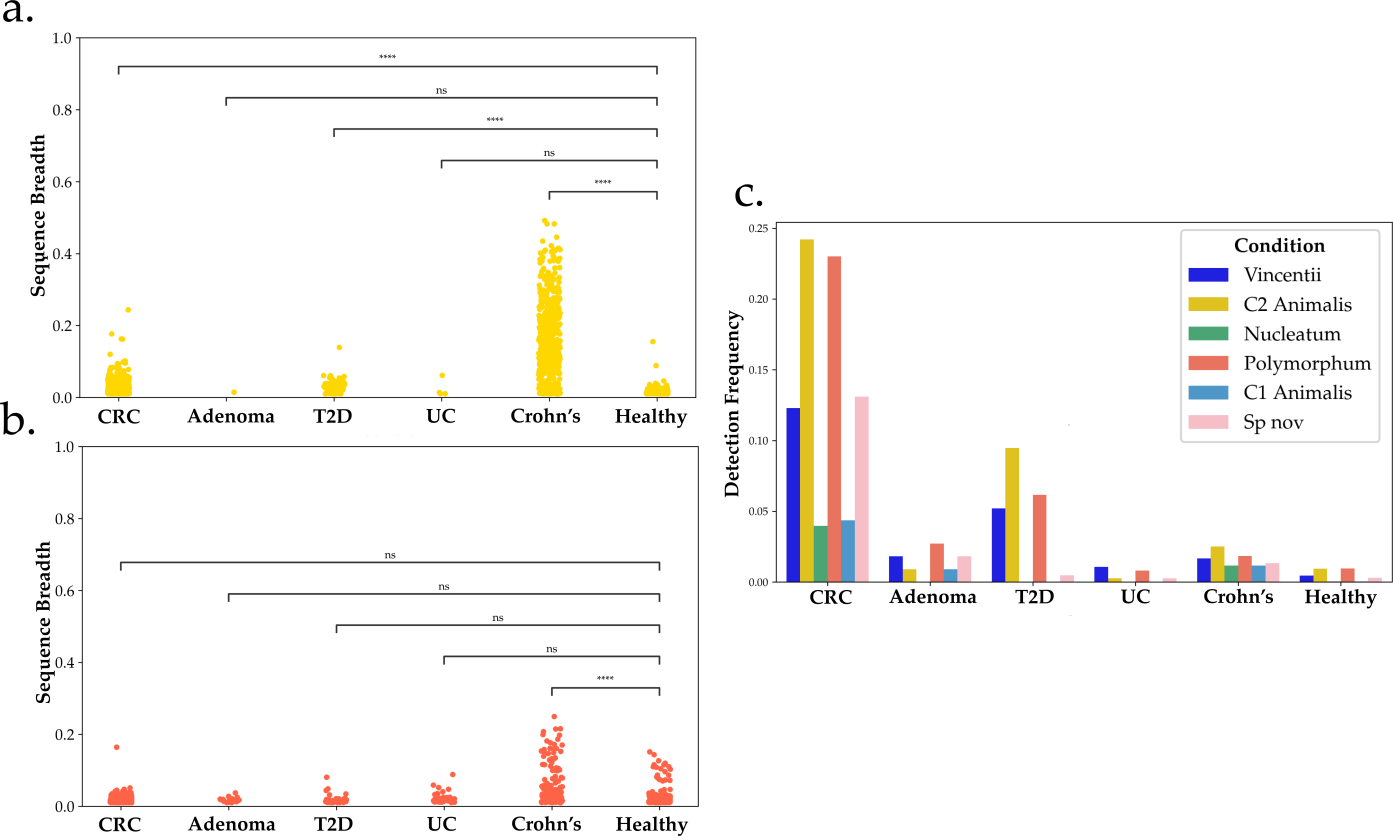
Sequence breadth and frequency differences in *F. nucleatum* population from disease to health. A total of 5,840 stool metagenomes, from healthy and diseased hosts, were collected. The genomes were split into populations, and the sequence breadth of each was recorded. Sequence breadth was then graphed if above detection threshold. Sequence breadth distributions of the disease conditions were compared to healthy via Mann-Whitney *U* test and corrected for false discovery rate. (a) Sequence breadth for C2 *animalis*. (b) Sequence breadth for *polymorphum*. (c) Population detection frequencies by condition. *P < 0.05, ****P < 1e−04, after Bonferroni correction.

In metagenomes from CRC and T2D stool samples, only C2 *animalis* sequence breadth was significantly higher than in healthy stool (Mann-Whitney *U* test, *P* < 1e−04, Fig. 5a). No other population was significantly increased in these diseases save *nucleatum* in CRC (*P* < 0.01, Fig. 5a and b; Fig. S1). The frequency of all *Fn* populations, particularly C2 *animalis* and *polymorphum*, was significantly higher in CRC than in the healthy cohort (*P* < 0.05) (Fig. 5c).

### *Polymorphum*, C2 *animalis* significantly elevated in Crohn’s disease

The highest sequence breadth measurements in the data set were observed in samples from Crohn’s disease, where C2 *animalis*, *polymorphum*, *vincentii*, and *Sn* were all significantly elevated (Fig. 5a and b; Fig. S1). In ulcerative colitis samples from the same data set, overall sequence breadth was not significantly higher than in healthy, and only *vincentii* was significantly increased (Mann-Whitney *U* test, *P* < 4.429e−04). Despite the high sequence breadth, detection frequency in Crohn’s disease was only modestly higher than in healthy samples, though statistically significant, while frequency in CRC was significantly higher than in Crohn’s disease (*P* < 0.05) (Fig. 5c)

### Clinical patterns in populations

As mentioned above, the genomes analyzed came from multiple body sites and pathologies. Patient health data were available for some, but not all, of the genomes. Each population besides *Sn* was found in both the oral and gut environments (Table 1). Disease metadata was only available for 38 genomes in the data set, many of which were cultivated directly from tissue or stool samples from CRC (Tables 3 and 4). At least one isolate in all populations aside from *Sn* was found in CRC, and at least one isolate in all populations aside from *Sn* and C1 *animalis* was found in a dental infection. However, the clearest disease signal was in C2 *animalis*, for which 28 of the 52 isolates were derived from CRC tissue or stool samples.

**TABLE 3:** Body site metadata for genomes after population assignment

Population	Oral	Gut	Urogenital
*nucleatum*	12	3	1
C2 a*nimalis*	15	30	2
*polymorphum*	14	3	0
*vincentii*	7	9	0
C1 a*nimalis*	16	3	0
sp. nov.	4	0	0

**TABLE 4:** Disease metadata for genomes after population assignment

Population	Dental infection	CRC
*nucleatum*	1	2
C2 a*nimalis*	1	28
*polymorphum*	5	2
*vincentii*	2	3
C1 a*nimalis*	0	3
sp. nov.	0	0

### Sex affects *F. nucleatum* population distribution

To determine if there were broadly different distributions of *Fn* populations based on patient sex, we first compared the detection frequencies of *Fn* in the gingiva. There was no difference in these distributions (chi-squared test of independence, *P* = 0.2290) (Fig. S2a).

We next compared every stool sample, both healthy and diseased. There was no significant difference in distribution among the sexes (chi-squared test of independence, *P* = 0.1728). C2 *animalis* and *polymorphum* were the most commonly found in stool from both male and female patients. The frequency of detection was higher in the male data set than in the female data set for every population (Fig. S2b).

We next compared sequence breadth scores between male and female patients. Both C2 *animalis* and *polymorphum* were significantly increased in male patients as compared to female patients (Mann-Whitney *U* test, *P* < 6.747e−04). No other populations exhibited a significant difference (Fig. S3a).

To investigate sex distributions in health and disease, we separated the cohort into healthy and diseased patients. Males with Crohn's disease showed significantly higher sequence breadth measurements than females, again primarily due to C2 *animalis* (Mann-Whitney *U* test, *P* < 4.173e−41) (Fig. S3b). Taken together, these data suggest sex differences in gut *Fn* are salient in disease states.

### Gut *F. nucleatum* increases with age, disease

We then investigated how age might affect *Fn* presence in the gut. We separated patient age into five roughly equal bins and compared the frequency and sequence breadth of *Fn* between healthy and diseased patients in every group (Table 3). The frequency and distribution of *Fn* populations in different age cohorts resemble the pattern for stool overall. C2 *animalis* is the most common population found in both the diseased and healthy cohorts, and *Fn* is more common overall in the diseased stool (Fig. S4a).

For sequence breadth, the same pattern holds for each population, but there also seems to be a consistent increase in breadth as patient age increases. If we split the cohort into healthy and diseased patients, we see that detection in healthy patients remains relatively low into old age, while diseased patients continue to exhibit the sharp increase described above (Fig. S4b).

Overall, the age and sex data indicate that *F. nucleatum* is more highly detected in older, unhealthy males. However, the rank order of the bacterial populations is not affected by the age or sex of the patient cohort.

### Read depth is not correlated with sequence breadth

When comparing metagenomic studies from different publications, read depth is a potential confounder (32). Studies with very high or low coverage could give the misleading impression that a certain population or species is more or less common than it truly is. To investigate whether read depth was affecting our results, we evaluated the read depth and total breadth of all samples. As C2 was the most frequently detected population in most of our studies, we compared studies by mean and maximum C2 sequence breadth (Fig. S5).

We assessed the correlation between detection and read depth in our studies. In each case, the correlation was not significantly different from zero among our studies. Other publications have suggested a rough threshold of 15 million reads in order to confidently detect low-abundance populations in a sample. Three of our studies are below that threshold: Brito, 2019; Hannigan, 2017; and Ghensi, 2019. Ghensi is a gingival study, and *Fn* would not be considered low-abundance in that setting, explaining the high max and mean detection we see here. However, in Hannigan and Brito, we do not find a single positive *Fn* detection of any population.

These studies are on saliva and stool, where *Fn* would be expected to occur in low abundance. Hannigan includes samples from CRC patients. In the other CRC studies, *Fn* C2 occurs at a rate of 28–32%. This suggests that while there is not a noticeable effect of read depth across our data set as a whole, it may be influencing our results at the lower extreme.

### *F. nucleatum* populations are functionally differentiated by iron transport and biomolecule synthesis gene clusters

**Fig 6:**
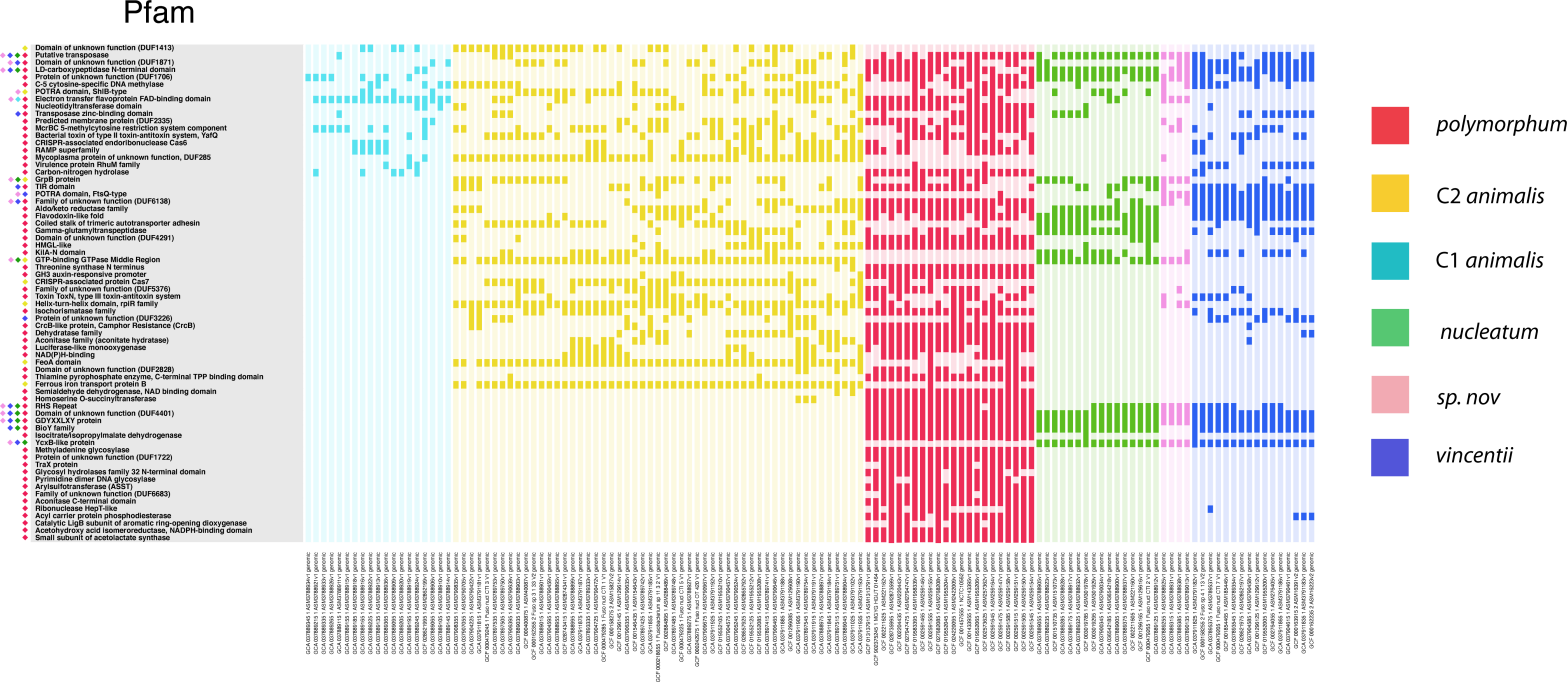
Functions enriched in populations. Gene clusters used to construct the pangenome were analyzed for enrichment among the populations via Rao’s test and corrected for false discovery rate. Clusters that were significantly enriched (left, vertical) and present in a majority of the genomes from at least one population were plotted with a colored bar. Markers at left indicate which populations each gene cluster is enriched in. Genomes (bottom) were colored according to HGT population assignment: light blue: C1 *animalis*, yellow: C2 *animalis*, red: *polymorphum*, green: *nucleatum*, pink: sp. nov, blue: *vincentii*.

To determine which functions distinguished populations, we performed a functional enrichment analysis on the gene clusters found in the pangenome, again using Pfam classifications for the clusters (20, 33). Based on the results of our metagenomic analyses, we specifically investigated C2 *animalis*, *polymorphum*, and *vincentii*. We placed a special emphasis on these three populations because of their different distributions in the gut and mouth; *vincentii* mainly being found in the mouth, C2 *animalis* being the most prevalent in the gut, and *polymorphum* appearing in both environments. We cataloged all clusters that were significantly enriched after FDR correction (*P* < 0.01) and present in a majority of the target population’s genomes (Fig. 6).

The results showed that *polymorphum* had the most specifically enriched gene clusters. Many of these 37 genes were involved in the synthesis of amino acids, fatty acids, and nucleic acids. C2 *animalis* had 11 specifically enriched genes, including ferrous iron transport proteins A and B, a trimeric autotransporter adhesin (involved in helping pathogens adhere to host cells), and several proteins whose function has not been fully characterized (34). *vincentii* had only seven specifically enriched gene clusters, four of which have not been fully characterized functionally. Examining clusters enriched in both *polymorphum* and *vincentii* uncovered 14 Pfams, including a biotin importer, a hydrolase, proteins involved in transposases, and more functionally uncharacterized proteins.

## DISCUSSION

Here, we show that the human body houses at least six populations of *Fn*, and that *Fn* population structure has robust implications for human health phenotypes. Our results, supported by HGT clustering and pangenomics, demonstrate differences in *Fn* population distribution across different diseases and clinical and demographic axes. Our work improves the specificity of classification for *Fn*, as most publications refer to four at most, and some refrain from acknowledging population diversity entirely (1, 8, 35).

Gene cluster comparisons of *Fn* populations indicate that each population has a distinct functional profile. Our analysis identified genes that were significantly enriched and present in a majority of only our target population and thus captures functions that help explain a population’s phenotype and physical biogeographic preferences. C2 *animalis*, which appears to be most salient in disease, was most enriched in iron transporters FeoA and FeoB. In some pathogenic bacteria, the Feo system has been identified as a key virulence factor. Feo mutation has been shown to prevent pathogens from colonizing the intestine (36). Iron also has been repeatedly shown to be salient in CRC (37). FeoA/B gene clusters were found in all but one C2 genome, but were completely absent from every other population aside from *polymorphum*, where they were found in 2 of the 17 genomes. We note that *polymorphum* had the second-highest detection in the gut environment; the presence of FeoA/B could be used as a biomarker in oral bacteria for ability to colonize the gut. C2 *animalis* was also highly enriched in two phage-related proteins, including Cas7 and an RpiR helix-turn-helix domain, indicating that phages may play a role in shaping *Fn* population structure.

*polymorphum* was enriched in more gene clusters than any of the other populations, the majority of which were involved in amino acid synthesis. Recent papers have shown that high concentrations of biomolecule synthesis gene clusters can provide metabolic independence, which allows bacterial populations to be more resilient to environmental perturbation, supporting a scenario in which *polymorphum* is well suited to inhabit multiple body sites (38). The most significantly enriched cluster, found in all *polymorphum* genomes, was CrcB, a fluoride exporter. Given fluoride’s preeminence in dental hygiene, it is tempting to connect *polymorphum*’s ability to clear fluoride to its dominance in the oral niche, as has been suggested in oral *Streptococci* (4, 39).

Like *polymorphum*, *vincentii* was found to be more prevalent than C2 *animalis* in the oral environment. We contrasted those proteins enriched in *vincentii* alone with those enriched in both *polymorphum* and *vincentii*. Most gene clusters from both categories were functionally uncharacterized, preventing us from drawing strong conclusions about functions favorable to the oral environment. However, both groups contained virulence proteins and phage remnants, again suggesting the importance of phages in shaping population structure. Finally, oxidative stress response proteins were common to both *polymorphum* and *vincentii*, suggesting that some aerotolerance may improve fitness in the gingival environment.

The HGT-based population assignments indicated that some genomes previously classified as *nucleatum* formed a separate network, a clade which was also visible in the core genome phylogeny (Fig. 1). To our knowledge, this is the first report of this new population. Metagenomic analyses indicated that this group, termed *sp. nov*., was roughly as common as C2 *animalis* in the gingiva. A population this common could play significant roles in gingival communities and merits further investigation.

Our simulation-based approach allows us to sensitively and confidently detect *Fn* in metagenomes (26). We report detection cutoffs of 14.2% for the oral microbiome and 1% for the gut microbiome. That the gut cutoff is so much lower than the oral can be explained by the abundance of *Fusobacterium* in the latter environment and a paucity in the former (29, 30). *Fn* is phylogenetically distant from most gut-resident organisms, and no members of the *Fusobacteriota* phylum are typically found in the gut (39). This means that there is very little false positive signal for *Fn* in stool metagenomes.

Our metagenomic results demonstrate that the distribution of *Fn* populations varies substantially from mouth to gut. In particular, we have shown that C2 *animalis* is found disproportionately in the gut compared to what one would expect from the mouth, especially in CRC, and that the rate C2 is found in CRC is greater than in the gingiva generally. *Fn* has previously been demonstrated to accelerate tumor growth, increase metastatic progression, and most recently, to increase tumorigenesis in mouse models (40–43). C2 is found much more frequently in CRC than any other population, but is only found in a minority of patient gingival samples. This finding raises the question of whether people who carry C2 *animalis* orally may be at higher risk of CRC.

To our knowledge, no study has specifically examined *Fn* species distributions in the oral microbiota of CRC patients. qPCR studies have been conducted on overall *Fn* load in CRC patient saliva, but our results suggest that these analyses are sharply limited by failing to consider subspecies (44, 45). Furthermore, we find that the detection of *Fn* in saliva is much more limited than in the gingiva, suggesting that any future *Fn* studies should prioritize gingival samples. Comparing paired gingival and stool samples from CRC patients and healthy donors would reveal if the carriage rate of C2 *animalis* is higher in the gingiva of CRC patients than in the general population; if so, C2 *animalis* could potentially be used as an oral biomarker for CRC risk.

We found that sequence breadth measurements for *Fn* in Crohn’s disease were higher than in any other stool samples we analyzed. This was true for both C2 *animalis* and *polymorphum*. Intriguingly, the frequency of *Fn* populations in Crohn’s samples was only slightly higher than in healthy samples, a discrepancy not explained by read depth. *Fn* is known to effectively colonize abscesses in the oral setting (4). Crohn’s patients suffer from intra-abdominal abscesses in 10–30% of cases (46). A previous study isolated oral bacteria from more than 75% of intra-abdominal abscesses (47). It is therefore possible that the high sequence breadth of *Fn* in Crohn’s metagenomes reflects patients with abdominal abscesses.

There are known to be sex differences in the gut microbiome, but their exact nature is still being determined, owing to the difficulty of separating sex from other confounders and the dearth of studies focusing specifically on sex differences in the microbiota (48, 49). We show that overall *Fn* is higher in males than females, though the population distribution is the same. In both the stool and the gingiva, the frequency of *Fn* detection was higher in males than in females, and in the stool, sequence breadth of C2 *animalis* and *polymorphum* was significantly higher in males than females. Together with the known higher risk of CRC for men and the documented association of *Fn* with cancer, further studies are warranted to understand the interactions between CRC, sex, and *Fn* (43, 50, 51).

Our results emphasize the need for holistic study of the human microbiota, with consideration at genome-level resolution and comparison across varied disease states and body sites. In the case of *Fn*, we have shown that subpopulation-level diversity cannot be ignored in human health, and that ecologically grounded approaches can help uncover commonalities between seemingly disparate disease states. Collapsing distinct bacterial populations into a single taxon could cause researchers to miss clinically relevant patterns of occurrence and distribution.

## MATERIALS AND METHODS

### Access and retrieval of genomes

All available *F. nucleatum* genomes at the scaffold level or above were downloaded from GenBank on 4 April 2024, via the python package ncbi-genome-download. This amounted to 133 genomes in total. These were used for all of our analyses. A full list of genomes is available in Table S1.

### Core genome phylogeny

A core genome phylogeny was created using PhaME 1.0.3. The tree contained the genomes above, in addition to one *F. periodonticum* genome provided as an outgroup (ATCC 33693, GCF_000160475.1). PhaME was configured to use ANI-based reference, paired reads, with both FastTree and RAxML, with a linear alignment cutoff of 0.1. Tree was rooted at the midpoint and visualized using Interactive Tree of Life (52).

### Creation of HGT networks

The genomes accessed above were processed according to the protocol set out by Arevalo et al. (14). First, all genomes were aligned pairwise using mugsy (53). Each alignment was then assessed for regions of 100% identity. An expected distribution of 100% identical regions was created based on the overall percent identity of the two genomes. This expected distribution was then compared to the expected distribution via the sum of squares. The resulting length bias score was used to draw edges between genomes in a network. If the lower bounds of the score’s 95% CI were greater than 0, an edge was drawn between the pair, as longer-than-expected identical regions are markers of HGT. The networks were then visualized via Cytoscape 3.8.0, using edge-weighted force-directed layout.

### Pangenome construction

The 133 genomes used to construct the HGT network were deposited in a single .fa file. In order to remove identical genomes, the rmdup command from the seqkit package was used (54). After deduplication, a 131-genome reference file remained. Using the Anvi'o metagenomics suite v7.1, this reference file was then built into a contigs database (default settings) (21). Features in the contigs database, including gene clusters, were identified using anvi-run-hmms on default settings. Gene clusters were then annotated with anvi-run-pfams, which draws on the Pfam (now InterPro) database (20).

### Empirical establishment of detection cutoffs

For each population, a detection cutoff was established using imGLAD (26). Two reference sets were created, one using genomes of common stool bacteria and one of common gingival bacteria, sourced from the human oral microbiome database (available in Tables S3 and S4) (55). For each environment, 1,000 simulated metagenomes were created using ART-MountRainier-2016-06-05 (56): 500 negative controls, consisting solely of reads from the reference set, and 500 positive, which included reads from both the reference set and the target *Fusobacterium* population. Target sequence breadth values were calculated for all simulated metagenomes and paired with positive and negative identities. These data were used to train a logistic regression. The result was the sequence breadth score needed for 95% CI of the target’s presence in a sample. These detection cutoffs were used to determine the presence of *Fusobacterium* populations in study metagenomes. In the gingival environment, the reference set included genomes representing each *Fn* population. In the stool, the reference set did not include *Fusobacteriota*, but the measured sequence breadth was examined in non-target population genomes. Figure 3a features the maximum off-target detection with a C2 *animalis* target genome.

### Access and retrieval of metagenomes

9,560 metagenomes were sourced from 11 studies, with priority given to those available in the CuratedMetagenomicData database (57). Metagenome accessions were obtained either via that database or via the NIH SRA Run Selector. Once located, metagenomes were downloaded via sra-tools v3.0.0, using prefetch and fasterq-dump. Metagenomes were stored and analyzed as uncompressed fastq files. When possible, metadata were sourced from CuratedMetagenomicData (57). Metagenomes were sourced from the following accessions: PRJNA398089, PRJNA217052, PRJNA422434, PRJNA48479, PRJEB49206, PRJNA389927, PRJNA763023, PRJEB10878, PRJNA255922, PRJEB7774, and PRJNA547717. Metadata for each metagenome included are available in Table S2.

### Alignment of metagenomes to reference genomes

Metagenomic samples were then aligned against the deduplicated reference file (see “Pangenome construction”) via bowtie2, with --sensitive and --no-unal enabled. The resulting sam files were then converted to bam using samtools and filtered at quality 1 (58, 59). This threshold was chosen because it disposed of low-quality alignments while still allowing multireads. With a database of such closely related bacteria, we expected to have many true multireads, so thresholds above 1 would have excluded authentic data. The bam files were then sorted and indexed via samtools.

### Age and sex profiles of cohort

Sex and age confounders are known to exist in the microbiome (48, 49, 60). In our data set, sex data were available for 9,097 metagenomes, 5,538 of which were stool metagenomes. Of those, 51.92% were from female patients, and 48.08% were from male. Numeric ages were available for 7,750 samples, 5,158 of which were stool metagenomes. We separated the ages into five categories, recorded in Table 5 below. There do not appear to be differences in the ages available for each sex (Fig. S6). Disease-derived samples were older on average than healthy samples.

**TABLE 5:** Age data for stool MGs

Age group	*n*	Percentage
0–19	1,057	20%
19–33	1,037	20%
33–49	1,002	19%
49–60	1,225	24%
60–89	837	16%
Total	5,158	

### Calculation of sequence breadth in alignments

Sorted bam files were analyzed via Anvi'o metagenomics suite (v7.1) (21). One merged profile database was created for each study. Detection scores for each split in a genome were then extracted and averaged for that genome. The presence of each population in a given metagenome was established via comparison with the detection cutoffs determined above. If any genome in a population was found to be above the cutoff, the population was considered to be present in that sample. All sequence breadth scores above cutoff were stored and plotted. All scores below cutoff were assigned a value of 0. Counts of populations present were divided by sample size in order to obtain frequencies. The count tables were compared via chi-squared tests while the scores were compared via scipy.stats (v.1.3.1) (61). Sequence breadth measurements were compared via Mann-Whitney *U* tests and scipy.stats. All of the above was accomplished using a Python script, which is available on GitHub (https://github.com/kellylab/sequence_breadth_tools/).

### Functional enrichment analysis

Functional enrichment analysis of reference genomes was performed using anvi-compute-functional-enrichment-across-genomes of the Anvi'o metagenomics suite (v7.1) (21, 33). A logistic regression was fitted to each gene function occurrence, using HGT population as the explanatory variable and functions from COG2020. A Rao score test was performed on each function, and *q* values were calculated for the test *P* values. *q* Values below 0.05 were considered to be significantly enriched. Significantly enriched genes were then filtered to exclude those not present in a majority of any population to maximize the generalizability of our findings.

## Data Availability

Metagenomes and genomes used in this paper are all available on public repositories; full lists of accessions are available in Tables S1 and S2.

## SUPPLEMENTAL MATERIAL

Supplemental figures mbio.02989-24-s0001.pdf Fig. S1-S6.

Supplemental tables mbio.02989-24-s0002.xlsx Tables S1-S4.

ASM does not own the copyrights to Supplemental Material that may be linked to, or accessed through, an article. The authors have granted ASM a non-exclusive, world-wide license to publish the Supplemental Material files. Please contact the corresponding author directly for reuse.
