# Supplementary material for: The physical biogeography of Fusobacterium nucleatum in health and disease
Source: mBio. 2025 Mar 10;16(4):e02989-24. doi: 10.1128/mbio.02989-24 (PMC11980382; doi:10.1128/mbio.02989-24)
Supplement: Supplemental figures — Fig. S1-S6. [file mbio.02989-24-s0001.pdf]

Supplemental Figures

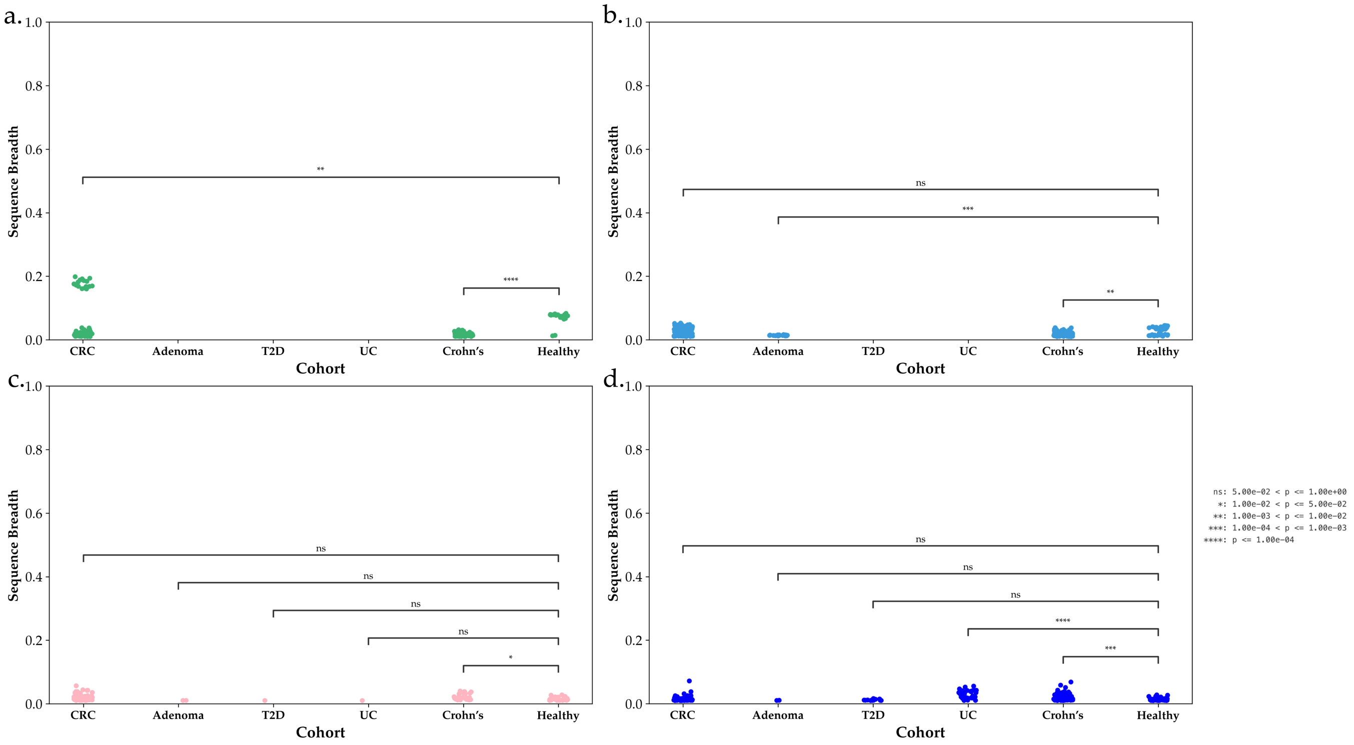

<u>Supplemental Figure 1: Few clear disease associations in other *Fn* populations</u>

A total of 5,840 stool metagenomes, from healthy and diseased hosts, were collected. The genomes were split into populations, and the sequence breadth of each was recorded. Sequence breadth was then graphed if above detection threshold. Sequence breadth distributions of the disease conditions were compared to healthy via Mann-Whitney U test and corrected for false discovery via Bonferroni. a) Sequence breadth of *nucleatum* across conditions. b) *C1 animalis* c) *sp. nov* d) *vincentii*.

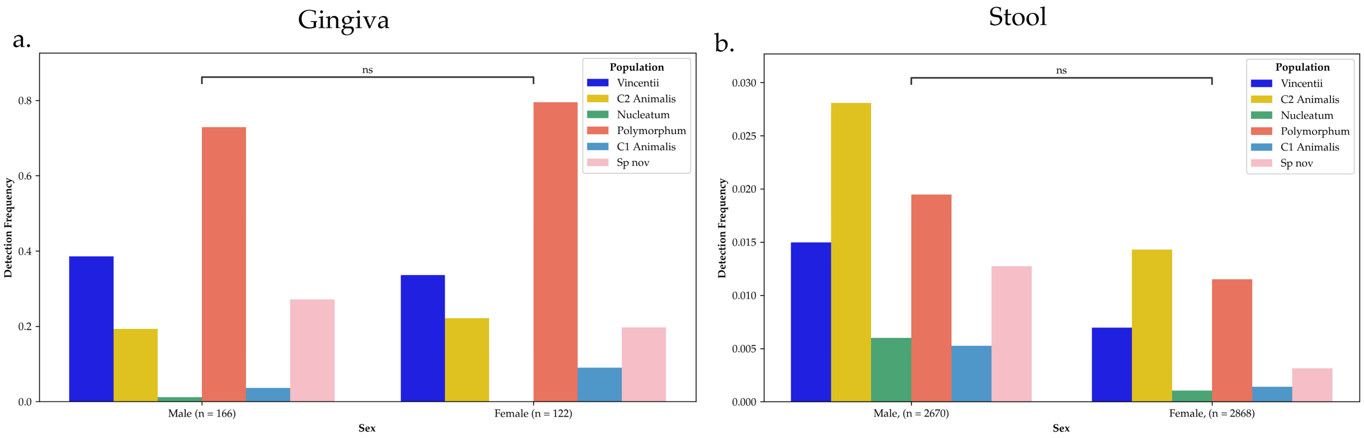

<u>Supplemental Figure 2: Sex has No Effect on *F. nucleatum* Population Distribution</u>

Gingival and stool samples were divided by sex. The detection frequencies in each group were established using sequence breadth thresholds gleaned from metagenomic simulations (0.1429 and 0.01, respectively). The distributions of populations in each group were compared via chi-squared test of independence. a. Comparison of populations in gingival samples by sex. b. Comparison of populations in stool samples by sex.

a.

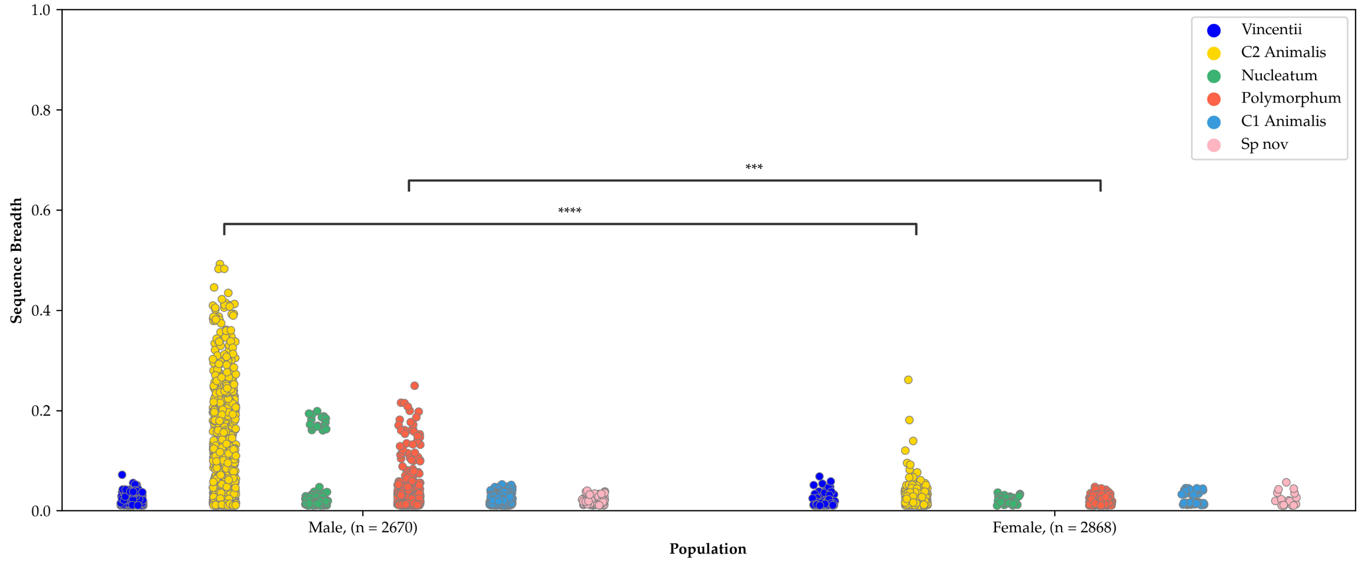

b.

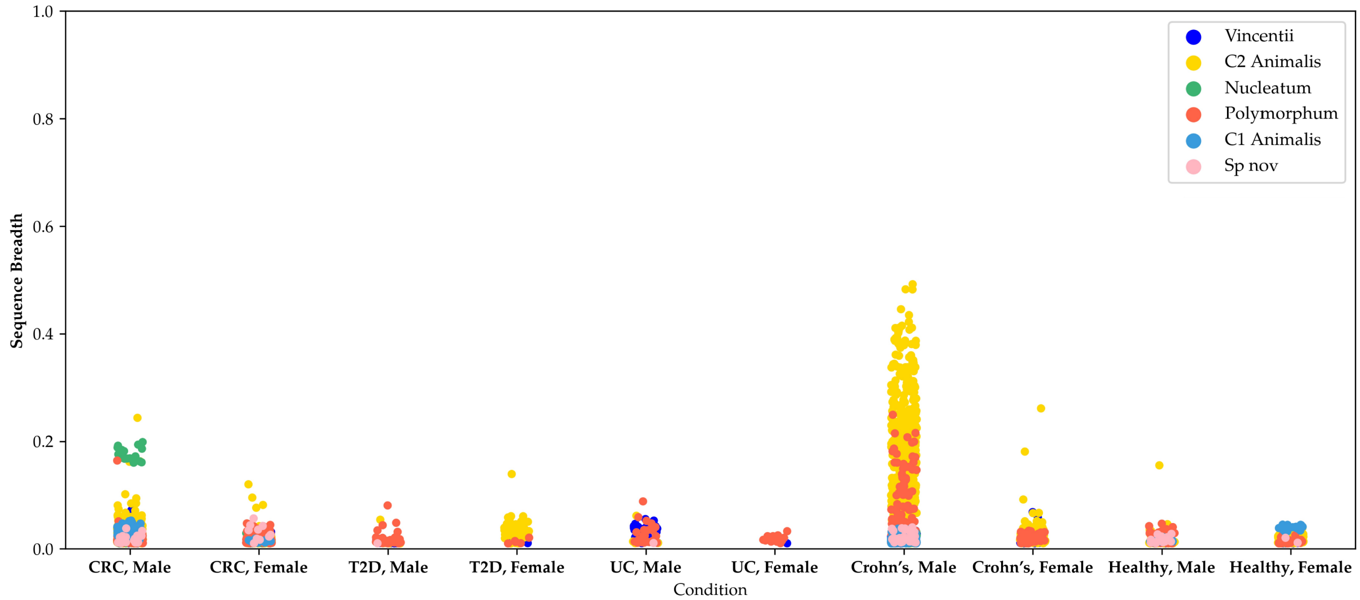

<u>Supplemental Figure 3: Increased C2 *animalis* and *Polymorphum* Detection in Males</u>

Stool metagenomes were split on the basis of sex and host disease. Sequence breadth of each *Fn* population was calculated. Groups were compared via Mann-Whitney U test. *** = p < 0.001. **** = p < 0.0001. a. Comparison of *Fn* populations in all stool metagenomes by sex. b. Comparison of *Fn* populations in CRC and healthy stool by sex. c. Visualization of *Fn* sequence breadth by disease, populations overlaid.

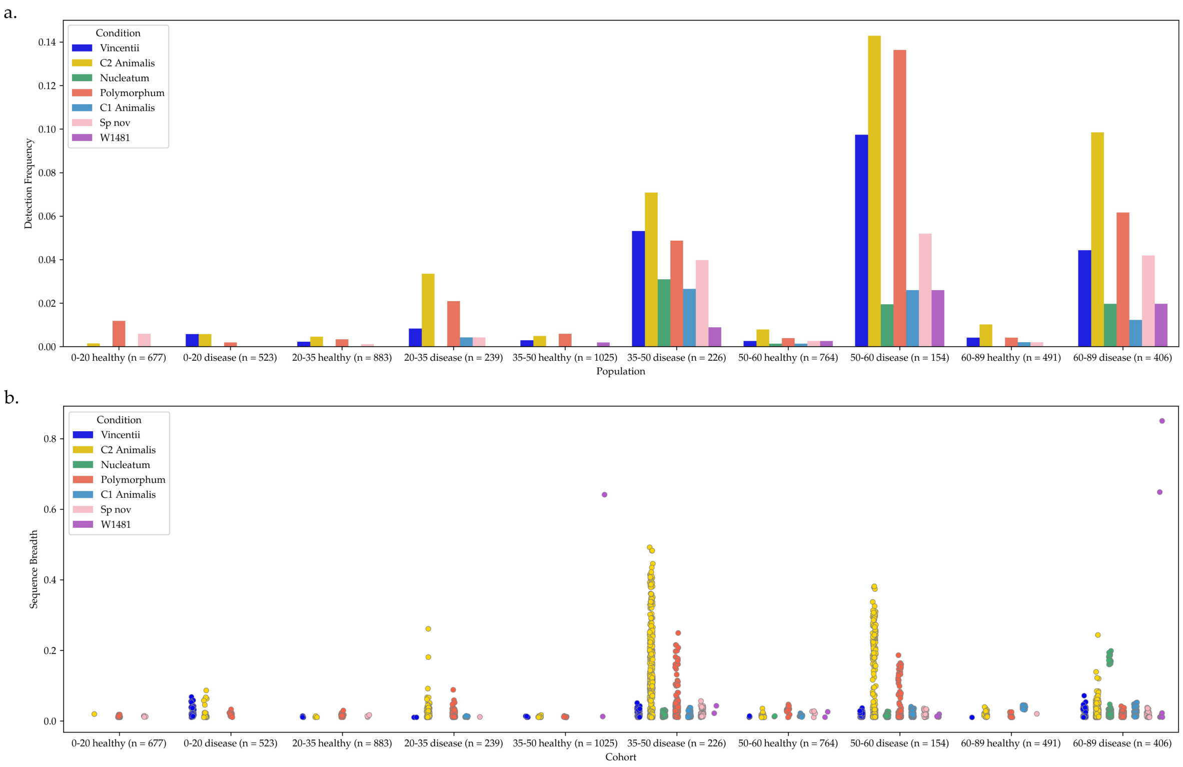

Supplemental Figure 4: Disease Status Dominates Age in *F nucleatum* Detection

Stool metagenomes for which a numeric age was available (4,711 in all) were separated into 5 roughly equal bins, and by health/disease status. Disease group included samples from patients with CRC, type 2 diabetes, ulcerative colitis, Crohn's disease, and adenoma. Sequence breadth for each *Fn* population in each sample was calculated and plotted if above detection threshold.

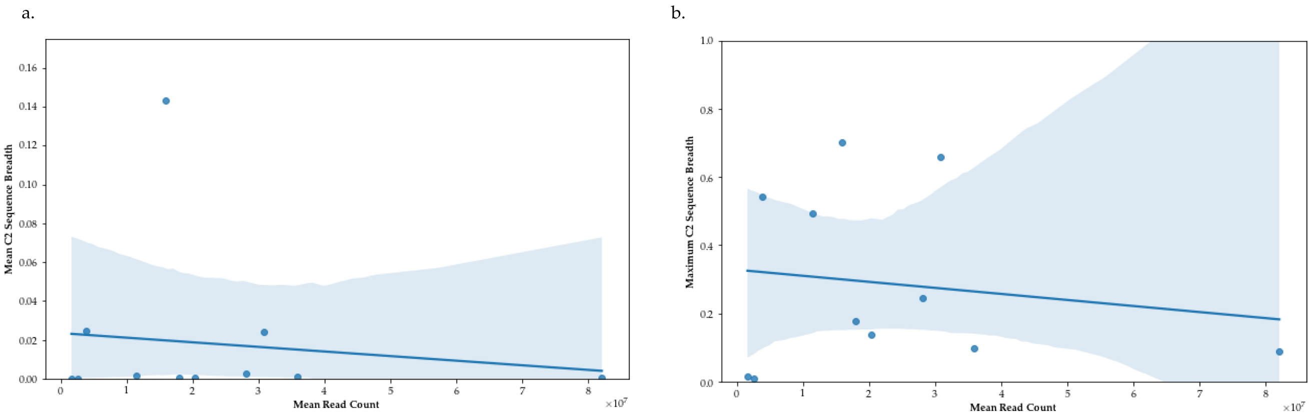

Supplemental Figure 5: Read Depth Has No Effect On Detection Trends

For each metagenomic study in our dataset, the number of reads for every sample was recorded, then the average read depth per study was calculated and plotted against a. the mean sequence breadth of C2 *animalis* and b. the maximum sequence breadth of C2 *animalis*. The relationship between sequence breadth and read depth was assessed via linear regression. In both cases, the correlation was not significantly different from zero ($p = 0.71$, $p = 0.67$, respectively).

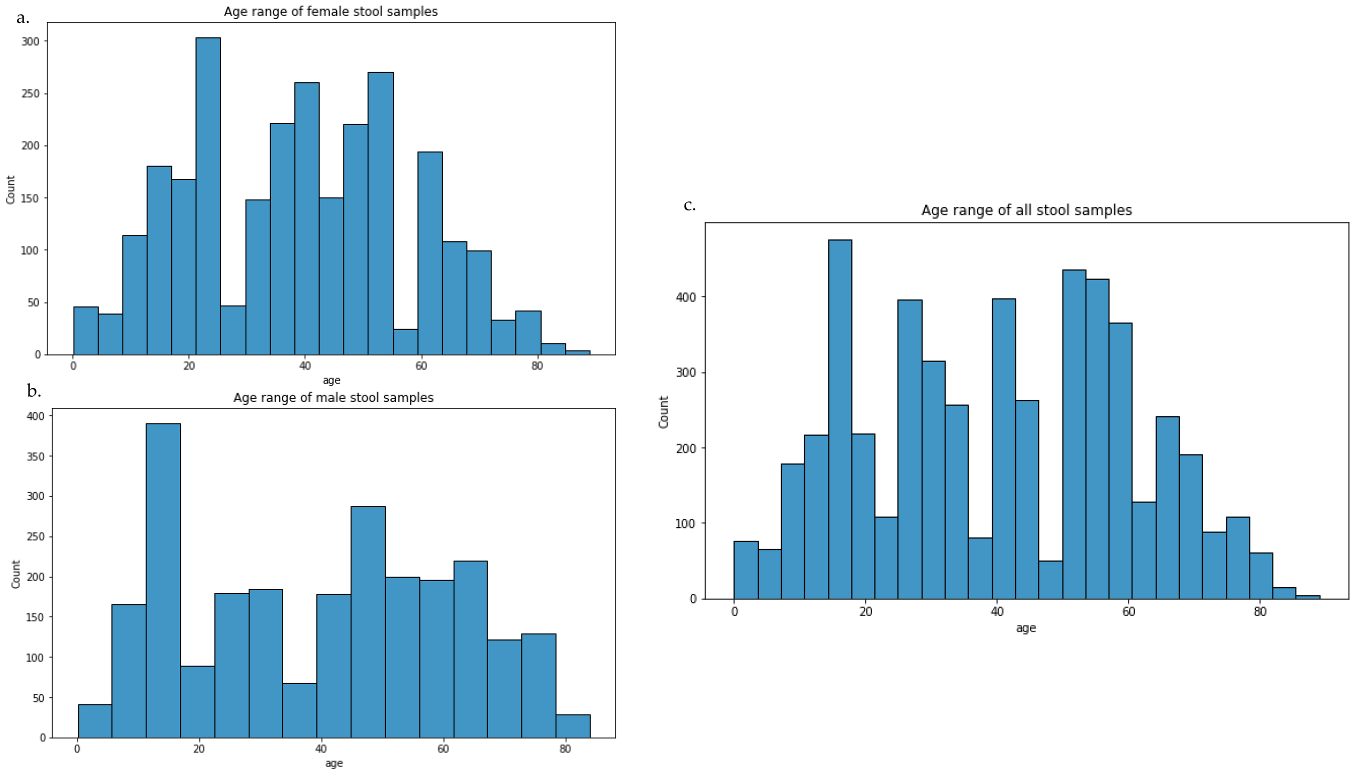

Supplemental Figure 6: Cohorts are Balanced Along Age and Sex Lines

Histogram of age and sex for patients where both were available. 51.92% of patients were female and 48.08% were male.
